# Relationships between developmental strategies for additional indications and price revisions for anticancer drugs in Japan

**DOI:** 10.1186/s12913-021-07360-w

**Published:** 2021-12-11

**Authors:** Hideki Maeda, Ayano Okabe, Kenichi Sakakura, Daniel Bin Ng, Manabu Akazawa

**Affiliations:** 1 Department of Regulatory Science, Meiji Pharmaceutical University, 2-522-1, Noshio, Kiyose, Tokyo, 204-8588 Japan; 2 Department of Public Health and Epidemiology, Meiji Pharmaceutical University, 2-522-1, Noshio, Kiyose, Tokyo, Japan; 3 Daiichi Sankyo Co., Ltd., 3-5-1, Nihonbashi-honcho, Chuo-ku, Tokyo, 103-8426 Japan; 4 Department of Health Economy Outcome Research, Astellas Pharma Global Development, Singapore, Singapore; 5 Department of Pharmacy Systems, Outcomes and Policy, University of Illinois at Chicago College of Pharmacy, Chicago, Illinois USA

**Keywords:** Anticancer drug, Oncology, Drug price revision, Additional indications, Japan, National Health Insurance drug price

## Abstract

**Background:**

The relationships between developmental strategies for additional indications and drug price revisions have not been thoroughly studied. Here, we investigated the price revisions for anticancer drugs approved in Japan.

**Methods:**

The study was based on published information on anticancer drugs approved between January 2009 and March 2020 in Japan. We investigated the relationships between the pharmacological and regulatory characteristics of anticancer drugs and occurrence/non-occurrence of the Japanese National Health Insurance (NHI) price revisions.

**Results:**

Eighty-one new anticancer drugs were given NHI price listings during the survey. On April 1, 2020, the prices of 23 anticancer drugs had been revised from the initial pricing, the prices were reduced for 21 drugs (91.3%). Several parameters showed the relationships between drug characteristics and NHI price revisions. The achievement of additional indications and compound type were identified as explanatory factors for these relationships. Additional indication profiles were defined to assess the relationships between the methods for additional indication achievement and price revisions. When the type of additional indication was “Expansion”, the percentage of drugs received NHI price revisions was the highest (*P*<0.001).

**Conclusions:**

NHI price revision was significantly related to the achievement of additional indications and compound type. The strategy for additional indications was found to affect the occurrence/non-occurrence of NHI price revisions.

**Supplementary Information:**

The online version contains supplementary material available at 10.1186/s12913-021-07360-w.

## Background

High drug prices are a major societal issue. In Japan, population aging is progressing more rapidly than that in other countries, and the national budget proportion associated with medical costs is increasing continuously [1]. Furthermore, Japan’s healthcare system involves universal insurance coverage. Accordingly, more than 90% of approved new molecular entities are listed in the National Health Insurance (NHI) system and are thus eligible for NHI reimbursement [2]. Thus, Japan’s problems with drug prices and the NHI are more severe than those in other countries. Moreover, the NHI pricing method in Japan is unique and highly complex. The initial pricing is, in principle, performed using one of the following methods: (i) the cost-accounting method and (ii) the similar-efficacy comparison method, involving comparison with one or more drugs with similar pharmacological activities [3]). The issue is not settled with drug price calculation, because the price is reassessed once every 2 years, often resulting in NHI price revision, whereby the price is usually reduced. Furthermore, from 2021, drug price reassessment will be carried out annually [4]. The reason for price reassessment is that drugs are bought and sold between pharmaceutical wholesalers and medical institutions or pharmacies at a price lower than the NHI price, thus, the basis of the regular NHI price revision is to lower the NHI price accordingly. There are also several special rules, such as market expansion repricing and, price reduction for a long-listed drug to be considered while revising drug price. These are all measures to control medical costs in Japan (Online Resource 1). In other words, Japan’s drug pricing system is different from that of other countries. It is characterized by a unique method of calculating the initial NHI price and the NHI price continues to decrease post-marketing [1–3].

There are many cancer types and patient strata for which treatment is unsatisfactory, and there is a need for new anticancer drugs. Anticancer drugs are often approved for indications of orphan cancers, and their novelty often results in high pricing because of premium rewards [5]. Generally, the clinical development of an anticancer drug is for one cancer type, and often, the subsequent development strategy is to achieve additional indications after approval for the initial cancer indication [6, 7]. Additionally, consideration is generally given to unmet medical needs and patient access to new drugs, and even if profitability is disregarded, cancer types are prioritized [7]. Therefore, in many cases, the return on the investment of anticancer drugs is expected to include not only sales for the initial indication, but also for additional indications. In the above process, drug price reduction in Japan is a hurdle to be overcome in establishing development strategies. At the first glance, increasing medical costs and new drug innovation are incompatible in some areas [8]. However, it is crucial to achieve a proper balance, and the calculation of appropriate drug prices enables the funding of research and development costs. Furthermore, it provides resources for the development of innovative drugs. The drug pricing system is important, and the aim should be to balance patient access and innovation funding [9–11]. Given this context, when establishing development strategies for anticancer drugs in Japan, including additional indications, it is crucial to consider NHI pricing methods, especially NHI price revision. However, these methods remain to be under-evaluated. In the present study, we investigated the relationships between NHI price revision and pharmacological and regulatory characteristics and development strategies, represented by additional indication achievement methods, for anticancer drugs approved in Japan.

## Methods

### Drug selection for cancer treatment

This was a retrospective survey of anticancer drugs approved in Japan between January 2009 and March 2020. Anticancer drugs are defined as therapeutic drugs that directly target malignant tumors. Hence, drugs to treat cancer-related pain, benign tumors, or pre-cancer lesions; palliative care drugs; diagnostic drugs; drugs used before anticancer drug administration; and prophylactic drugs for adverse effects were excluded.

### Data collection

Information was collected from publicly available data. Information regarding applications for approval was mostly obtained from the Pharmaceuticals and Medical Devices Agency’s website [12]. Information related to NHI prices and revisions was obtained from the Central Social Insurance Medical Council’s website [13], Ministry of Health, Labour and Welfare’s medical insurance website [14], *NHI Drug Price Standards* [15], and *NHI Drug Price Standards Quick Reference Tables* [16]. The pharmacological and regulatory characteristics of each anticancer drug were assessed, and an independent database was prepared. The data included the following: (i) generic name; (ii) submission-related information; (iii) administration route; (iv) therapeutic indication classification code; (v) indications; (vi) cancer type, in terms of the number of patients, as follows: major: gastric, lung, colorectal, hepatic, breast, and prostate cancer; orphan: cancer with orphan diseases designation received from Ministry of Health, Labour and Welfare in Japan.; and minor: neither major nor orphan; (vii) additional indication-related information; (viii) approval conditions (conditional approval, post-marketing all-case surveillance); (ix) guideline for proper clinical use of the drug; (x) initial NHI price-related information; (xi) sales predictions; (xii) compound type; and (xiii) company. This study was conducted according to the Strengthening the Reporting of Observational Studies in Epidemiology (STROBE) reporting guidelines [17] for cross-sectional studies.

### Classification of methods for additional indication achievement

Methods for additional indication achievement can be classified as follows (for details of the classification, please refer to Online Resource 1):

A. *Expansion*: The number of patients with an additional indication is greater than that with the initial indication.
B. *Retention*: The number of patients with an additional indication that is same as that with the initial indication.
C. *Targeting orphan cancers*: The additional indication is an orphan cancer or the patient population for it is smaller than that for the initial indication.

### Occurrence/non-occurrence of NHI price revision

We surveyed all NHI price revisions in Japan and compared the results before and after each revision for each drug. Whether NHI price revision occurred is defined as follows:

(i) *No revision*: The price changes because of a consumption tax increase or is lower than 3% based on market price or similar assessments at the time of each revision.
(ii) *Revision occurred*: The conditions in (i) do not apply and/or NHI price revisions are made based on special rules.

The NHI price revision is based on two major methods. One is regular price revision by a regular drug price survey, and the other is based on special rules for price revision. The special rules include premium rewards for innovative development, price reduction for long-listed drugs, market expansion-related repricing, dosage/regimen change-related repricing, repricing for indication change, and other calculations at the time of repricing for orphan drugs, pediatric indications, and genuine clinical usefulness (Online Resource 2).

### Statistical methods

Statistical analysis were performed using Microsoft® EXCEL and JMP, with a significance level of 5%. To evaluate the relationships between pharmacological characteristics and NHI price revision, Pearson’s χ^2^ test was used to compare nominal variables and Student *t*-test was used to compare continuous variables. The Kaplan–Meier analysis was used for the time course of NHI price revision. Multivariable logistic regression was used to analyze the potential factors associated with NHI price revision.

## Results

### Characteristics of anticancer drugs with NHI price listing

**Fig. 1:**
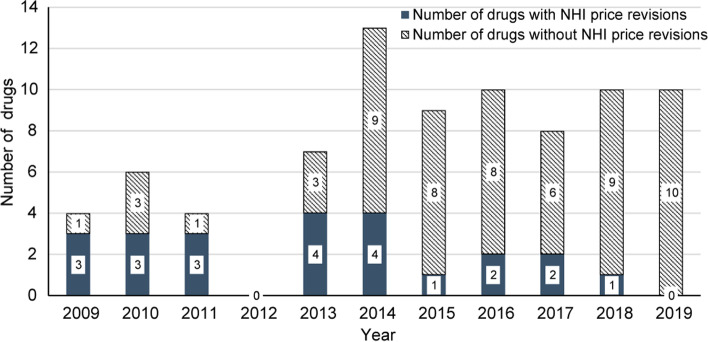
Number of oncology drugs in the National Health Insurance price list and price revisions by year

Totally, 153 indications of anticancer drugs were approved in Japan between January 2009 and March 2020, including 81 initial approvals and 72 with additional indications. All 81 approved new anticancer drugs had NHI price listings. The number of anticancer drugs with NHI price listings each year, classified by occurrence/non-occurrence of NHI price revision, is shown in Fig. 1. The mean number of anticancer drugs with NHI price listing per year was 7.4 (range: 0–13). Investigation of the occurrence/non-occurrence of NHI price revision for the 81 new anticancer drugs by April 1, 2020, showed revision and retention of the initial price for 23 (28.4%) and 58 (71.6%) drugs, respectively. Of the 23 drugs, the number of drugs with price revisions for different reasons, with some revisions for two or more reasons, were as follows: revision based on regular price revision, 13 (56.5%); market expansion-related repricing, 14 (60.7%); premium rewards for orphan disease indication, 4 (17.4%); and dosage/regimen change-related repricing, 3 (13.0%). For the following two drugs, the NHI price was increased, both of which are cases of premium rewards for orphan disease achieved as an additional indication: (i) ibrutinib: from ¥9,367 to ¥10,135 (increase by 8.2%), and (ii) eribulin: from ¥64,070 to ¥67,121 (increase by 4.8%). For the other 21 drugs, the NHI price was reduced; the mean reduction rate for the 23 drugs was 14.1%.

The pharmacological and regulatory characteristics of the 81 new anticancer drugs surveyed are shown in Table 1.

**Table 1:** Characteristics of compounds and regulatory programs of 81 oncology drugs

Characteristic		Number	Percentage
Compound type	Molecularly targeted drug	43	53.1%
	Antibody	15	18.5%
	Cytotoxic drug	11	13.6%
	Hormonal drug	6	7.4%
	Immunotherapy drug	5	6.2%
	Other	1	1.2%
Indication	Hematologic tumor	31	38.3%
	Solid tumor	50	61.7%
Dosage form	Oral	43	53.1%
	Injection	38	46.9%
Number of approvals for additional indication	None	50	61.7%
	1	13	16.0%
	2	12	14.8%
	3	1	1.2%
	4	2	2.5%
	5	2	2.5%
NHI initial drug package price	<1,000 yen	2	2.5%
	<10,000 yen	34	42.0%
	<100,000 yen	25	30.9%
	<1,000,000 yen	19	23.5%
	>1,000,000 yen	1	1.2%
Calculation system for NHI price standard	Cost accounting method	25	30.9%
	Similar efficacy comparison method (I)	51	63.0%
	Similar efficacy comparison method (II)	3	3.7%
	Inter-specification adjustment	2	2.5%
Corrective premium rate	With premium rate	57	70.4%
	None	24	29.6%
NHI price revision	Yes	23	28.4%
(as of April 1, 2020)	No	58	71.6%
Number of NHI price revisions	None	58	71.6%
	1	18	22.2%
	2	3	3.7%
	3	1	1.2%
	4	0	0.0%
	5	1	1.2%
Type of pharmaceutical company	Japanese domestic company	32	39.5%
	Foreign company	49	60.5%
Review period (days)	Average ± SD	73.3 ± 44.1	
	Maximum	289	
	Minimum	21	

*NHI* National Health Insurance, *SD* Standard Deviation

### Relationships between anticancer drug characteristics and NHI price revision

The relationships between the occurrence/non-occurrence of NHI price revision and the pharmacological and regulatory characteristics of anticancer drugs were investigated (Table 2).

**Table 2:** Characteristics of drugs and NHI price revision

Characteristic		Without price revision		With price revision		Total		*P-*value
Total		58	71.6%	23	28.4%	81	100.0%	-
Indication	Solid tumor	32	55.2%	18	78.3%	50	61.7%	0.003
	Hematologic tumor	26	44.8%	5	21.7%	31	38.3%	
Additional indication	Yes	17	29.3%	15	65.2%	32	39.5%	0.003
	No	41	70.7%	8	34.8%	49	60.5%	
Initial indication	Rare cancer	35	60.3%	8	34.8%	43	53.1%	0.046
	Normal cancer	24	41.4%	15	65.2%	39	48.1%	
Initial indication	Major Cancer	18	31.0%	10	43.5%	28	34.6%	0.297
	Normal cancer	40	69.0%	13	56.5%	53	65.4%	
Drug	Drug	57	98.3%	23	100.0%	80	98.8%	0.526
	Regenerative medicine	1	1.7%	0	0.0%	1	1.2%	
Sakigake designation (Breakthrough therapy designation)	Yes	1	1.7%	0	0.0%	1	1.2%	0.526
	No	57	98.3%	23	100.0%	80	98.8%	
Conditional approval	Yes	1	1.7%	0	0.0%	1	1.2%	0.526
	No	57	98.3%	23	100.0%	80	98.8%	
Priority/Expedited review	Yes	7	12.1%	5	21.7%	12	14.8%	0.269
	No	51	87.9%	18	78.3%	69	85.2%	
Post-marketing all-case surveillance	Yes	40	69.0%	11	47.8%	51	63.0%	0.076
	No	18	31.0%	12	52.2%	30	37.0%	
Guideline for proper clinical use of the drug	Yes	2	3.4%	3	13.0%	5	6.2%	0.106
	No	56	96.6%	20	87.0%	76	93.8%	
Dosage form	Oral	26	44.8%	12	52.2%	38	46.9%	0.550
	Injection	32	55.2%	11	47.8%	43	53.1%	
Novelty of the drug	First in class	13	22.4%	6	26.1%	19	23.5%	0.725
	Other	45	77.6%	17	73.9%	62	76.5%	
Compound type	Molecularly targeted drug	33	56.9%	10	43.5%	43	53.1%	0.025
	Hormonal drug	2	3.4%	4	17.4%	6	7.4%	
	Antibody	14	24.1%	1	4.3%	15	18.5%	
	Cytotoxic drug	6	10.3%	5	21.7%	11	13.6%	
	Immunotherapy drug	2	3.4%	3	13.0%	5	6.2%	
	Other	1	1.7%	0	0.0%	1	1.2%	
NHI pricing method	Similar efficacy comparison method (I)	39	67.2%	12	52.2%	51	65.4%	0.057
	Similar efficacy comparison method (II)	3	5.2%	0	0.0%	3	3.8%	
	Cost accounting method	16	27.6%	9	39.1%	25	32.1%	
	Inter-specification adjustment	0	0.0%	2	8.7%	2	2.6%	
Corrective premium rate	Yes	41	70.7%	16	69.6%	57	73.1%	0.920
	No	17	29.3%	7	30.4%	24	30.8%	
Sales ranking of the company	Top 10 in Japan	22	37.9%	6	26.1%	28	34.6%	0.312
	Other	36	62.1%	17	73.9%	53	65.4%	
Type of pharmaceutical company	Japanese domestic company	24	41.4%	8	34.8%	32	39.5%	0.584
	Foreign company	34	58.6%	15	65.2%	49	60.5%	
Peak sales amounts (Oku-yen)		86.4 ± 115.2		102.3 ± 120.3		90.9 ± 116.9		0.585
From application to approval (days)		305.0 ± 88.9		402.6 ± 169.6		332.7 ± 125.6		<0.001
From approval to NHI price listing (days)		70.6 ± 39.6		80.1 ± 53.4		73.3 ± 44.1		0.387
From NHI price listing to date of analysis (days)		1415.3 ± 937.2		2510.8 ± 994.3		1726.4 ± 1074.1		<0.001

*NHI* National Health Insurance

Regarding the occurrence/non-occurrence of NHI price revision, stratified analysis was performed based on pharmacological and regulatory characteristics. Significant differences (*P* < 0.05) were observed in the presence/absence of additional indications, solid tumor versus hematologic tumor, initial indication, compound type, time from application for manufacturing approval until approval, and time since the initial NHI price listing date. In other words, anticancer drugs with NHI price revision were more likely to have additional indications, such as solid tumors, to have initial indications that were not orphan diseases, and, in terms of compound type, to be molecular-targeted drugs or immunotherapeutic drugs. On the contrary, anticancer drugs without NHI price revision were more likely to have a short time from application for manufacturing approval until approval and since the initial NHI price listing.

**Fig. 2:**
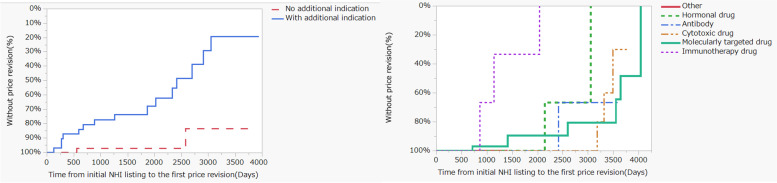
Kaplan-Meier curve for National Health Insurance price revisions by stratification of drug characteristics. Left panel: Stratified by additional indication, *P* < 0.001, log-rank test, Median time for an additional indication: 2410 days. Right panel: Stratified by compound type. *P* < 0.001, log-rank test, Median time for hormonal agent: 3050 days, Median time for cytotoxic drug: 3484 days, Median time for molecularly targeted drug: 3638 days, Median time for immunotherapy drug: 1141 days

**Fig. 3:**
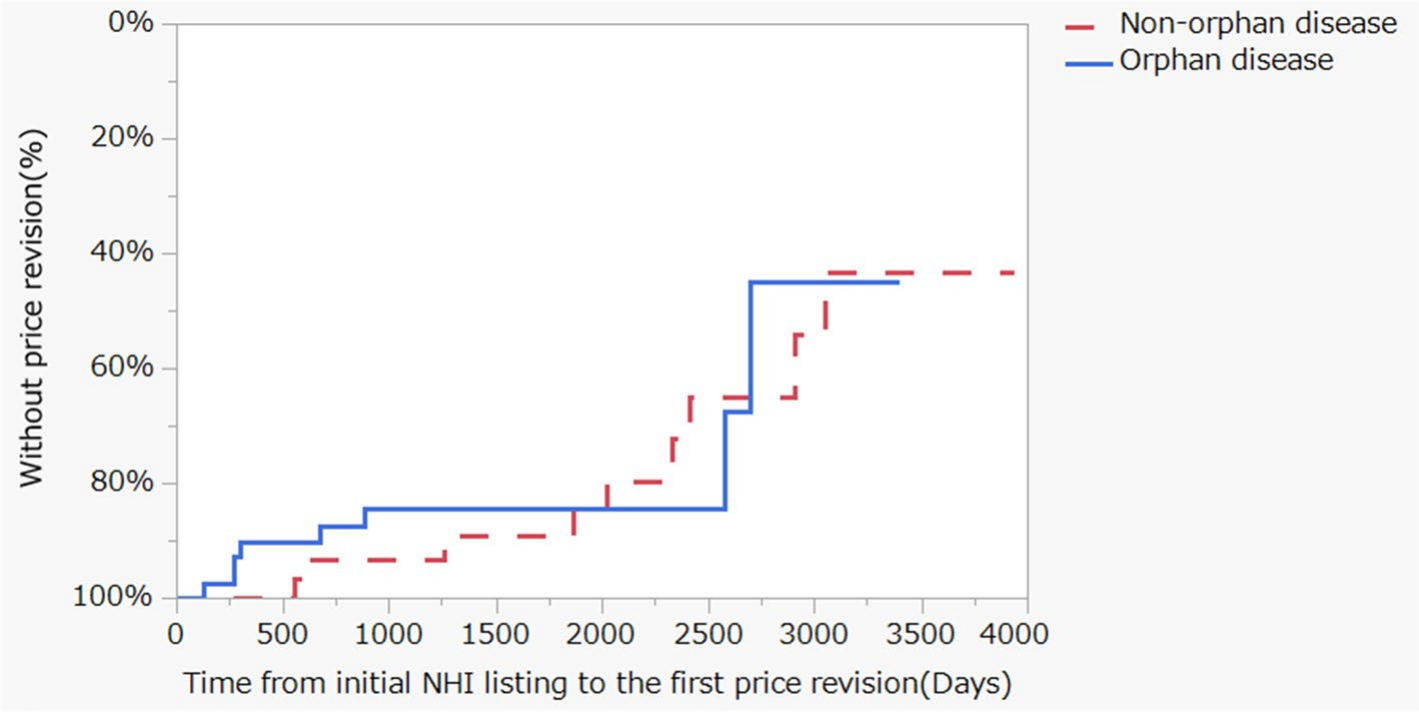
Kaplan-Meier curve for National Health Insurance price revisions by stratification of drug characteristics. Stratified by initial indication: Orphan or non-orphan cancer. *P* = 0.7682, log-rank test, Median time for an orphan cancer: 2695 days, Median time for a non-orphan cancer: 3051 days

**Fig. 4:**
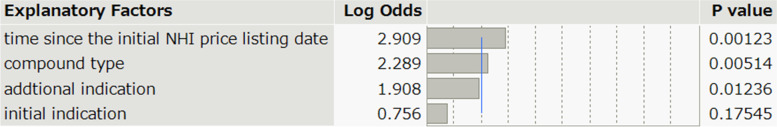
Multivariable logistic regression analysis for National Health Insurance price revisions. Factors were selected by stepwise method (variable increase/decrease method, threshold: *p* < 0.25). Test for the whole model: *p* < 0.001, *R*^2^ = 0.4358. Compound type is classified into molecularly targeted drug, hormonal drug, antibody, cytotoxic drug, immunotherapy drug and other. Additional indication is classified into yes (with additional indication) and no (no additional indication). Initial indication is classified into rare cancer, minor cancer and major cancer. NHI: National Health Insurance

The Kaplan–Meier analysis was used to investigate the changes with time in the nominal variables with significant differences, as shown in Table 2. There were significant differences in additional indications and compound types (Fig. 2). The same method was used to investigate the 35 drugs for which the initial indication was an orphan cancer, and no significant differences were found (Fig. 3). To examine the contribution of the explanatory factors presented in Table 2 to the NHI price revision, we conducted multivariable logistic regression analysis. The results of this analysis showed that three explanatory factors of “time since the initial NHI price listing date”, “compound type”, and “additional indications” were found to have large contributions (Fig. 4).

### Relationships between the type of procedure for additional indications and NHI price revisions

The occurrence/non-occurrence of NHI price revision in different development strategies is shown in Table 3.

**Table 3:** Relationship between the type of procedure for additional indications and NHI price revisions

	Without NHI price revision		With NHI price revision		Total	*P-*value*
Without additional indication	41	83.7%	8	16.3%	49	<0.001
Type A (Expansion)	7	36.8%	12	63.2%	19	
Type B (Retention)	8	88.9%	1	11.1%	9	
Type C (Targeting small cancer)	2	50.0%	2	50.0%	4	
Total	58	71.6%	23	28.4%	81	

*NHI* National Health Insurance

* Pearson’s chi-square test

Type-A drugs (expansion) had more often undergone NHI price revision (*P* < 0.001). For type-A drugs, the mean revised drug price was 88.2% before revision, whereas the means for type-B and type-C drugs were 102.8 and 99.5%, respectively, indicating that there was almost no change on average for these drugs.

## Discussion

### NHI drug pricing system and price revision system

In this study, we investigated the relationships between NHI price revisions and anticancer drug characteristics over time. The results suggest that the achievement of additional indications and compound type are significant explanatory factors (Table 2, Fig. 4). Considering the rules of NHI price revision in Japan, it is natural that drugs with a longer period of time since the NHI price calculation are more likely to be subject to NHI price revision, but the additional indications and compound type were suggested by our study. Furthermore, although future quantitative investigations are needed, a development strategy with cancer affecting a small number of patients as the initial indication, followed by one or more major cancers as additional indications, will result in the highest drug price reduction in the Japanese drug pricing system. The NHI pricing method used in Japan is unique and complex [1, 18]. In principle, for the initial pricing, cost-accounting and similar-efficacy comparison methods are used [3]. Among the drugs priced using the latter, premium rewards are added to some, such as for innovation and/or usefulness. These include orphan disease drugs and drugs for pediatric use and/or *sakigake* (i.e., accelerated approval of drugs designated as breakthrough therapies and that address unmet medical needs) [19, 20]. Furthermore, revisions may include price adjustment for consistency with overseas prices, inter-specification adjustments, and different dosage forms [21]. This system is relatively non-transparent, because the process involves decisions made after repeated negotiations between pharmaceutical companies and governmental agencies.

The health technology assessment (HTA) performed in all developed countries has no more than a supplementary role within the Japanese drug pricing system [22]. In Japan, HTA was only introduced after a prolonged debate [23]. It was first performed at a pilot scale, and then introduced full-scale [24, 25]. Thus, it is not used when calculating drug prices; it is merely used to supplement judgment about the appropriateness of drug prices in the Japanese system. Furthermore, in the Japanese drug pricing system, the same price is not retained once it has been calculated, and the price may be reduced based on market price assessments, which are performed once every 2 years and are to be performed annually from 2021. In addition to NHI price revisions based on market prices, prices may be reassessed according to special rules, and preliminary investigations of the approach for drug price reduction have been performed [26, 27]. These special rules are usually applied for market expansion-related repricing and price reduction for drugs listed long term. Price reduction is sometimes performed due to orphan diseases as additional indications [25], and it involves top-down decision-making, centered on the Central Social Insurance Medical Council, and without negotiations with pharmaceutical companies. Similar to the drug pricing method, the process is also non-transparent.

### NHI price systems in other countries

Efforts to balance cost containment and oncology drug access are not unique to Japan. In 2018, China proposed a volume-based procurement program to optimize drug pricing [28, 29]. Furthermore, the National Reimbursement Drug List was formally established in 2000. It covers 52% of China’s population under government urban health insurance programs and serves as a means of drug price negotiation for high-cost drugs [30]. In contrast to re-pricing in Japan, renegotiation and re-pricing of drugs occurs at either the end of the 2-year contract duration or the addition of new indications for reimbursement. Considering likely pricing pressure and market dynamics, the re-pricing often results in significant price reduction [29].

Alternatively, Korea was one of the first Asian countries to mandate pharmacoeconomic data submission for reimbursement decision-making. In Korea, reimbursement assessments and price negotiations are mandatory for new drugs. While cost effectiveness, as assessed by the Health Insurance Review of Assessment Service, is used to determine reimbursement, prices are fixed through negotiations with the National Health Insurance Service. Importantly, Korea has also implemented three methods to improve patient access to high-cost drugs: risk-sharing agreements, essential drug designation, and a waiver for cost-effectiveness analysis [31]. In the case of drug post-listing re-pricing, expanded indications also often trigger a post-listing price-cutting [32, 33].

In several Western European countries, re-pricing is often triggered by either a new product entry or an expansion of the indications. However, in many instances, a more robust evaluation requiring an updated dossier and economic models is required. In these cases, the results of re-pricing are often not clear, and there is a significant variability in re-pricing although the most frequent result is price reduction [3].

The expansion of indications for an anticancer drug often resulting in re-pricing is consistent among China, Korea, and Western European countries. However, the level of detail and the focus of the evaluation process have some variabilities. Most re-pricing cases result in a price reduction and are driven by various factors such as market dynamics and the economic effect of additional volume.

### Discussion on results of the present study and difficulties related to appropriate NHI price revisions

In the present study on the Japanese drug pricing system, we focused on NHI price revision and factors such as the effects of drug pharmacological and regulatory characteristics, and development methods were investigated. There are several studies on factors related to premium rewards during initial drug pricing in Japan [34, 35]. However, to the best of our knowledge, this is the first study on the factors affecting NHI price revision for anticancer drugs, methods for additional indication achievement, and NHI price revision.

Regarding development strategies for anticancer agents, development for cancer types with major unmet medical needs is invariably considered first, and such cancer types are often orphan cancers. In the Japanese drug pricing system, typically, if the indication at the time of new drug approval is an orphan cancer, the NHI drug price is awarded a premium and subsequently not readily reduced. However, the findings of the present study do not support the hypothesis that drugs for orphan cancers are not readily subject to NHI price revision (Fig. 3) [36], suggesting that even in the case of orphan anticancer drugs, other factors lead to drug price reduction.

### Future issues and proposals

In 2018, clear standards were established for additional indication-related repricing for market expansion. These standards specify that market expansion-related repricing is applicable when market expansion due to additional indications results in sales of more than ¥35 billion per year. This change in the system was associated with the innovative anticancer drug nivolumab. Nivolumab, first developed in Japan, was approved for melanoma, an orphan cancer, and thus achieved a high drug price; thereafter, it was approved for non-small-cell lung cancer. Therefore, its sales increased rapidly, leading to a prompt reduction in the price by 50% as a matter of urgency [37]. We consider this price reduction to be irrational and excessive. The reasons for the significant differences based on the types of compounds, shown in Fig. 2, are that these drugs are affected by major reductions in the drug prices of similar compounds.

The changes in the drug pricing system in 2018 resulted in clear criteria for additional indication-related repricing for market expansion. However, there has been no change in the difficulty in predicting the sales associated with additional indications or the situations in which development costs can be recouped. Drug price reduction due to market expansion-related repricing has the potential to discourage innovative drug research and development [38, 39].

In the United States and European Union, an indication-based pricing system has been examined recently. In this system, a drug does not have a single price, but its price is calculated separately for each indication, according to its value for that indication [40–42]. If indication-based drug pricing were to be introduced in Japan, it would probably result in a more objective and transparent system. Furthermore, when developing anticancer drugs, development starting with orphan cancers, based on unmet medical needs, is a sensible approach. From the perspective of drug pricing system or NHI price revision, it is considered unacceptable to hinder patients’ access to innovative drugs. Thus, we consider indication-based drug pricing an appropriate system.

This study had some limitations. First, this was a retrospective survey using publicly available information. Second, the study involved drugs that were approved and had NHI price listings. Drugs whose development was discontinued and had not been approved were not included. Third, the classification of development methods was qualitative, based on the number of patients and principal cancer type. In future studies, it will be necessary to perform quantitative classification with the number of patients and sales as indices.

In conclusion, we found that the presence/absence of additional indications and compound type were significant factors for the occurrence/non-occurrence of NHI price revision. Furthermore, the NHI price revision was influenced by the strategies for additional indication achievement for anticancer drugs. If the initial indications were rare cancers and the additional indications were cancers affecting more patients, drug prices decreased. We consider indication-based drug pricing an appropriate system.

## Supplementary Information

**Additional file 1: Online Resource 1.** Types of methods for additional indications in the development strategies of anticancer drugs

**Additional file 2: Online Resource 2.** Summary of the method for drug price revision in Japan

## Data Availability

We originally built the dataset. Detailed information is presented in “2.2 Data collection”.

## Abbreviations

NHI: National health insurance
HTA: Health technology assessment
