# Supplementary material for: Relationships between developmental strategies for additional indications and price revisions for anticancer drugs in Japan
Source: BMC Health Serv Res. 2021 Dec 11;21:1329. doi: 10.1186/s12913-021-07360-w (PMC8665599; doi:10.1186/s12913-021-07360-w)
Supplement: Supplementary file 1 — Additional file 1: Online Resource 1. Types of methods for additional indications in the development strategies of anticancer drugs [file 12913_2021_7360_MOESM1_ESM.docx]

**Online Resource 1. Summary of the method for drug price revision in Japan**

| Classification | Item | Contents | Timing of review for price revision | Price  after revision |
| --- | --- | --- | --- | --- |
| Regular price revision | | Price revision based on drug price survey | Once every 2 years until 2020,  once a year from 2021 | ↓ |
| Special rules  for price revision | Premium rewards for  innovative development | Development of orphan drugs | Irregular, review at the  time of regular price revision | → |
|  |  | Development request by regulatory agency |  |  |
|  |  | Drugs at premium prices because of usefulness or innovation |  |  |
|  |  | First-in-class drug |  |  |
|  |  | Same mechanism of action as the first-in-class drug |  |  |
|  | Price reduction for  long-listed drugs | Additional price reduction to the  price of the generic drugs in the case of long-listed drugs | Ten years after the launch of  the generic drug | ↓ |
|  | Market expansion-related re-pricing | Price reduction based on market expansion significantly  beyond prior projections | Timing of NHI price listing  (4 times a year) | ↓ |
|  | Other re-pricing | Re-pricing for dosage/regimen changes Re-pricing for additional indications | Timing of NHI price listing  (4 times a year) | ↓ |
|  | Premium rewards at  regular price revision | Premium rewards for pediatric indications | Irregular, review at the  time of regular price revision | → or ↑ |
|  |  | Premium rewards for orphan disease indications |  |  |
|  |  | Premium rewards for true clinical benefits |  |  |

NHI: National Health Insurance
