# Supplementary material for: Relationships between developmental strategies for additional indications and price revisions for anticancer drugs in Japan
Source: BMC Health Serv Res. 2021 Dec 11;21:1329. doi: 10.1186/s12913-021-07360-w (PMC8665599; doi:10.1186/s12913-021-07360-w)
Supplement: Supplementary file 2 — Additional file 2: Online Resource 2. Summary of the method for drug price revision in Japan [file 12913_2021_7360_MOESM2_ESM.docx]

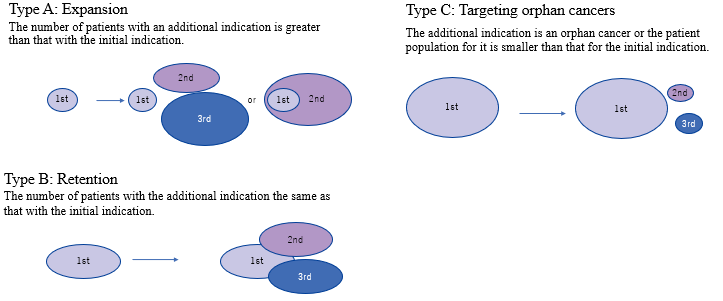

**Online Resource 1.** Types of methods for additional indications in the development strategies of anticancer drugs
